# *S*-Benzyl­thiouronium 4-anilinobenzene­sulfonate

**DOI:** 10.1107/S160053680802727X

**Published:** 2008-08-30

**Authors:** Hoong-Kun Fun, Suchada Chantrapromma, E. Deepak D’Silva, P. S. Patil, S. M. Dharmaprakash

**Affiliations:** a X-ray Crystallography Unit, School of Physics, Universiti Sains Malaysia, 11800 USM, Penang, Malaysia; b Crystal Materials Research Unit, Department of Chemistry, Faculty of Science, Prince of Songkla University, Hat-Yai, Songkhla 90112, Thailand; c Department of Studies in Physics, Mangalore University, Mangalagangotri, Mangalore 574 199, India; d Department of Physics, KLE Society’s KLE Institute of Technology, Gokul Road, Hubli 590 030, India

## Abstract

In the title compound, C_8_H_11_N_2_S^+^·C_12_H_10_NO_3_S^−^, the NH group of the *S*-benzyl­thiuronium is protonated and the inter­planar angle between the phenyl ring and the CH_2_—S=C(NH_2_)_2_ unit is 47.44 (10)°. In the 4-anilinobenzene­sulfonate anion, the inter­planar angle between the two rings is 44.07 (8)°. In the crystal structure, anions are linked into chains along the *c*-axis direction by N—H⋯O hydrogen bonds, while additional N—H⋯O inter­actions link the cations to the anions in chains along the *b*-axis direction. These chains are further inter­connected into a two-dimensional network parallel to the *bc* plane by C—H⋯O inter­actions. C—H⋯π contacts are also observed.

## Related literature

For bond-length data, see: Allen *et al.* (1987 ▶). For background to the applications of *S*-benzyl­thiuronium chloride and sodium diphenyl­amine-4-sulfonate, see, for example: Liao *et al.* (2004 ▶); Liu *et al.* (2006*a* ▶,*b* ▶); Mostafa (2006 ▶).
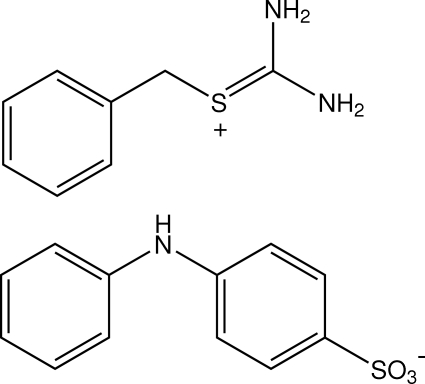


## Experimental

### 

#### Crystal data

C_12_H_10_NO_3_S^+^·C_8_H_11_N_2_S^−^
*M* *_r_* = 415.54
Monoclinic, 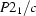

*a* = 14.4918 (4) Å
*b* = 9.2024 (2) Å
*c* = 16.3944 (4) Å
β = 113.529 (1)°
*V* = 2004.57 (9) Å^3^
*Z* = 4
Mo *K*α radiation
μ = 0.29 mm^−1^
*T* = 100.0 (1) K
0.24 × 0.07 × 0.03 mm

#### Data collection

Bruker SMART APEXII CCD area-detector diffractometer
Absorption correction: multi-scan (*SADABS*; Bruker, 2005 ▶) *T*_min_ = 0.879, *T*_max_ = 0.992
45812 measured reflections
5838 independent reflections
4320 reflections with *I* > 2σ(*I*)
*R*_int_ = 0.057

#### Refinement

*R*[*F* ^2^ > 2σ(*F* ^2^)] = 0.043
*wR*(*F* ^2^) = 0.107
*S* = 1.07
5838 reflections
337 parameters
All H-atom parameters refined
Δρ_max_ = 0.50 e Å^−3^
Δρ_min_ = −0.43 e Å^−3^

### 

Data collection: *APEX2* (Bruker, 2005 ▶); cell refinement: *APEX2*; data reduction: *SAINT* (Bruker, 2005 ▶); program(s) used to solve structure: *SHELXTL* (Sheldrick, 2008 ▶); program(s) used to refine structure: *SHELXTL*; molecular graphics: *SHELXTL*; software used to prepare material for publication: *SHELXTL* and *PLATON* (Spek, 2003 ▶).

## Supplementary Material

Crystal structure: contains datablocks global, I. DOI: 10.1107/S160053680802727X/sj2532sup1.cif

Structure factors: contains datablocks I. DOI: 10.1107/S160053680802727X/sj2532Isup2.hkl

Additional supplementary materials:  crystallographic information; 3D view; checkCIF report

## Figures and Tables

**Table 1:** Hydrogen-bond geometry (Å, °)

*D*—H⋯*A*	*D*—H	H⋯*A*	*D*⋯*A*	*D*—H⋯*A*
N1—H1*N*1⋯O1^i^	0.85 (2)	1.99 (2)	2.836 (2)	173.0 (18)
N2—H1*N*2⋯O3^ii^	0.92 (3)	2.04 (3)	2.9561 (19)	172 (3)
N3—H1*N*3⋯O1	0.809 (19)	2.015 (19)	2.8204 (19)	174 (2)
N2—H2*N*2⋯O3^iii^	0.839 (19)	2.015 (19)	2.8069 (19)	157.2 (19)
N3—H2*N*3⋯O2^ii^	0.91 (2)	1.96 (2)	2.8633 (17)	173 (2)
C9—H9⋯O1	0.97 (2)	2.463 (18)	2.8563 (18)	103.8 (13)
C19—H19*B*⋯O2^iv^	0.97 (2)	2.57 (2)	3.332 (2)	134.8 (14)
C4—H4⋯*Cg*2^v^	0.947 (19)	3.22 (2)	3.943 (2)	134.7 (14)
C17—H17⋯*Cg*1^iii^	0.99 (2)	2.92 (2)	3.471 (2)	116.0 (15)

Symmetry codes: (i) 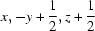; (ii) 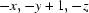; (iii) 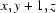; (iv) 
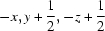
; (v) 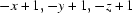. *Cg*1 and *Cg*2 are the centroids of C7–C12 and C13–C18 benzene rings, respectively.

**Fig. 1.**
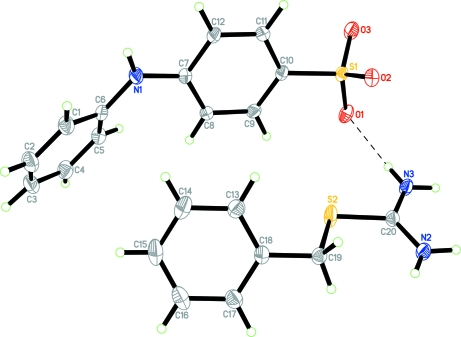
The asymmetric unit of (I), showing 50% probability displacement ellipsoids and the atomic numbering. The N—H···O hydrogen bond is drawn as a dashed line.

**Fig. 2.**
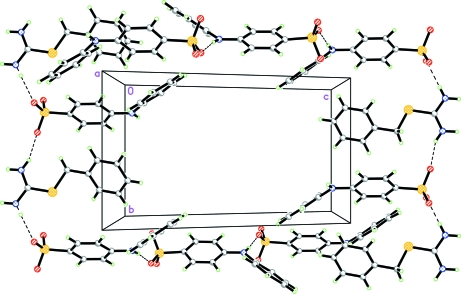
The crystal packing of (I), viewed along the a axis. Hydrogen bonds are drawn as dashed lines.

## supplementary crystallographic information

### Comment

*S*-benzylthiouronium chloride is a useful compound in pharmaceutical and biomedical science (Mostafa, 2006) and also in electrochemistry (Liao *et al.*, 2004) whereas sodium diphenylamine-4-sulfonate is extensively used in nanomaterial studies (Liu *et al.*, (2006*a*, *b*). Both compounds have the potential to form hydrogen bonds. As part of our investigations into solid state hydrogen bonding, the title compound (I) was synthesized and herein we report its crystal structure.

The molecular structure of the title compound consists of a C_8_H_11_N_2_S^+^ cation and a C_12_H_10_NO_3_S^-^ anion (Fig. 1). An NH group of the *S*-benzylthiouronium unit was protonated to become a NH_2_ moiety. Neither the cation and the anion are planar as can be seen from the interplanar angle between the C13–C18 benzene ring and the least-squares plane through the S2/C20/N2/N3 unit being 47.44 (10)°. In the diphenylamine-4-sulfonate anion, the interplanar angle between the the two benzene rings (C1–C6 and C7–C12) is 44.07 (8)°. The C13–C18 benzene ring makes dihedral angles of 71.72 (9)° and 29.45 (9)° with the C1–C6 and C7–C12 benzene rings, respectively. The cation is linked to the anion by an N—H···O hydrogen bond (Fig. 1). The conformation of the dimethylamino group with respect to the *S*-benzyl substituent is reflected in the torsion angles C20–S2–C19–C18 = -177.78 (11)° and C19–S2–C20–N2 = 14.60 (17)°. Bond lengths and angles in (I) are in normal ranges (Allen *et al.*, 1987).

In the crystal packing (Fig. 2 and Table 1), the anions are linked into chains along the *c* direction by N1—H1N1···O1 hydrogen bonds whereas the cations are linked with the anions into chains along the *b* direction by N2—H1N2···O3, N2—H2N2···O3  and N3—H2N3···O2 hydrogen bonds. These chains are further inter-connected into a two dimensional network parallel to the *bc* plane by C19—H19B···O2 interactions. C—H···π interactions were also observed in the  crystal (Table 1); Cg_1_ and Cg_2_ are the centroids of C7–C12 and C13–C18 benzene rings, respectively.

### Experimental

The title compound was synthesized by mixing solutions of the sodium salt of diphenylamine sulfonate (0.54 g) in distilled water (5 ml) with 5 drops of 1 M HCl and *S*-benzylthiouronium chloride (1.0 g) in distilled water (5 ml). The mixed solution immediately yields a precipitate in ice cold water. This was filtered and dried. Colorless block-shaped single crystals of the title compound suitable for *x*-ray structure determination were recrystallized from methanol by slow evaporation of the solvent at room temperature.

### Refinement

All H atoms were located in a difference map and were refined isotropically. The highest residual electron density peak is located at 0.86 Å from C10 and the deepest hole is located at 0.65 Å from S1.

### Crystal data

**Table:**

C_12_H_10_NO_3_S^+^·C_8_H_11_N_2_S^–^	*F*_000_ = 872
*M**_r_* = 415.54	*D*_x_ = 1.377 Mg m^−^^3^
Monoclinic, *P*2_1_/*c*	Mo *K*α radiation λ = 0.71073 Å
Hall symbol: -P 2ybc	Cell parameters from 5838 reflections
*a* = 14.4918 (4) Å	θ = 2.5–30.0º
*b* = 9.2024 (2) Å	µ = 0.29 mm^−^^1^
*c* = 16.3944 (4) Å	*T* = 100.0 (1) K
β = 113.529 (1)º	Block, colorless
*V* = 2004.57 (9) Å^3^	0.24 × 0.07 × 0.03 mm
*Z* = 4	

### Data collection

**Table:**

Bruker SMART APEXII CCD area-detector diffractometer	5838 independent reflections
Radiation source: fine-focus sealed tube	4320 reflections with *I* > 2σ(*I*)
Monochromator: graphite	*R*_int_ = 0.057
Detector resolution: 8.33 pixels mm^-1^	θ_max_ = 30.0º
*T* = 100.0(1) K	θ_min_ = 2.5º
ω scans	*h* = −20→20
Absorption correction: multi-scan(SADABS; Bruker, 2005)	*k* = −12→12
*T*_min_ = 0.879, *T*_max_ = 0.992	*l* = −23→23
45812 measured reflections	

### Refinement

**Table:**

Refinement on *F*^2^	Secondary atom site location: difference Fourier map
Least-squares matrix: full	Hydrogen site location: inferred from neighbouring sites
*R*[*F*^2^ > 2σ(*F*^2^)] = 0.043	All H-atom parameters refined
*wR*(*F*^2^) = 0.107	*w* = 1/[σ^2^(*F*_o_^2^) + (0.0444*P*)^2^ + 0.7114*P*] where *P* = (*F*_o_^2^ + 2*F*_c_^2^)/3
*S* = 1.07	(Δ/σ)_max_ < 0.001
5838 reflections	Δρ_max_ = 0.50 e Å^−^^3^
337 parameters	Δρ_min_ = −0.43 e Å^−^^3^
Primary atom site location: structure-invariant direct methods	Extinction correction: none

### Special details

**Table:**

Experimental. The low-temperature data was collected with the Oxford Cyrosystem Cobra low-temperature attachment.
Geometry. All e.s.d.'s (except the e.s.d. in the dihedral angle between two l.s. planes) are estimated using the full covariance matrix. The cell e.s.d.'s are taken into account individually in the estimation of e.s.d.'s in distances, angles and torsion angles; correlations between e.s.d.'s in cell parameters are only used when they are defined by crystal symmetry. An approximate (isotropic) treatment of cell e.s.d.'s is used for estimating e.s.d.'s involving l.s. planes.
Refinement. Refinement of *F*^2^ against ALL reflections. The weighted *R*-factor *wR* and goodness of fit *S* are based on *F*^2^, conventional *R*-factors *R* are based on *F*, with *F* set to zero for negative *F*^2^. The threshold expression of *F*^2^ > σ(*F*^2^) is used only for calculating *R*-factors(gt) *etc*. and is not relevant to the choice of reflections for refinement. *R*-factors based on *F*^2^ are statistically about twice as large as those based on *F*, and *R*- factors based on ALL data will be even larger.

### Fractional atomic coordinates and isotropic or equivalent isotropic displacement parameters (Å^2^)

**Table:**

	*x*	*y*	*z*	*U*_iso_*/*U*_eq_	
S1	0.08636 (3)	0.27987 (4)	0.16160 (2)	0.01695 (10)	
S2	0.19068 (4)	0.70226 (5)	0.20872 (3)	0.02907 (12)	
O1	0.15746 (9)	0.34329 (13)	0.12876 (7)	0.0216 (3)	
O2	−0.00092 (8)	0.37242 (12)	0.14360 (7)	0.0200 (2)	
O3	0.05615 (10)	0.13272 (12)	0.12701 (7)	0.0274 (3)	
N1	0.28844 (11)	0.27210 (17)	0.55470 (9)	0.0234 (3)	
N2	0.08033 (12)	0.85826 (16)	0.06346 (10)	0.0231 (3)	
N3	0.10880 (11)	0.61743 (16)	0.04555 (9)	0.0221 (3)	
C1	0.37263 (13)	0.4172 (2)	0.68520 (11)	0.0237 (4)	
C2	0.45695 (15)	0.4866 (2)	0.74499 (12)	0.0303 (4)	
C3	0.54646 (15)	0.4825 (2)	0.73360 (12)	0.0319 (4)	
C4	0.55080 (13)	0.4064 (2)	0.66248 (11)	0.0267 (4)	
C5	0.46709 (12)	0.33550 (19)	0.60270 (11)	0.0215 (3)	
C6	0.37628 (12)	0.34205 (18)	0.61244 (10)	0.0191 (3)	
C7	0.24732 (12)	0.27071 (17)	0.46289 (10)	0.0176 (3)	
C8	0.29225 (12)	0.33508 (18)	0.41073 (10)	0.0188 (3)	
C9	0.24368 (12)	0.33377 (18)	0.31869 (10)	0.0186 (3)	
C10	0.15065 (12)	0.26719 (16)	0.27700 (9)	0.0163 (3)	
C11	0.10566 (12)	0.19952 (17)	0.32809 (10)	0.0183 (3)	
C12	0.15354 (12)	0.20140 (18)	0.41962 (10)	0.0195 (3)	
C13	0.22213 (14)	0.71334 (19)	0.40844 (11)	0.0235 (4)	
C14	0.28471 (15)	0.6889 (2)	0.49686 (12)	0.0287 (4)	
C15	0.36132 (15)	0.7846 (2)	0.54192 (12)	0.0325 (4)	
C16	0.37589 (15)	0.9061 (2)	0.49858 (12)	0.0325 (4)	
C17	0.31450 (13)	0.9297 (2)	0.40940 (11)	0.0248 (4)	
C18	0.23738 (12)	0.83402 (18)	0.36394 (10)	0.0192 (3)	
C19	0.17094 (13)	0.85965 (18)	0.26715 (10)	0.0199 (3)	
C20	0.11899 (12)	0.73130 (18)	0.09651 (10)	0.0191 (3)	
H1	0.3074 (15)	0.419 (2)	0.6925 (13)	0.032 (5)*	
H2	0.4509 (15)	0.539 (2)	0.7926 (14)	0.041 (6)*	
H3	0.6056 (15)	0.527 (2)	0.7781 (13)	0.035 (5)*	
H4	0.6119 (15)	0.402 (2)	0.6544 (12)	0.029 (5)*	
H5	0.4718 (13)	0.2865 (19)	0.5562 (12)	0.020 (5)*	
H8	0.3557 (14)	0.384 (2)	0.4365 (12)	0.023 (5)*	
H9	0.2763 (13)	0.381 (2)	0.2840 (12)	0.022 (5)*	
H11	0.0404 (13)	0.1511 (19)	0.2983 (11)	0.015 (4)*	
H12	0.1223 (13)	0.156 (2)	0.4549 (12)	0.023 (5)*	
H13	0.1681 (15)	0.647 (2)	0.3794 (13)	0.029 (5)*	
H14	0.2756 (14)	0.609 (2)	0.5268 (13)	0.029 (5)*	
H15	0.4034 (17)	0.771 (2)	0.6006 (15)	0.042 (6)*	
H16	0.4294 (15)	0.973 (2)	0.5305 (13)	0.038 (6)*	
H17	0.3262 (14)	1.017 (2)	0.3794 (12)	0.034 (5)*	
H19A	0.1915 (13)	0.943 (2)	0.2439 (12)	0.024 (5)*	
H19B	0.1000 (14)	0.863 (2)	0.2565 (12)	0.023 (5)*	
H1N1	0.2478 (15)	0.245 (2)	0.5773 (13)	0.027 (5)*	
H1N2	0.0428 (18)	0.863 (3)	0.0029 (17)	0.058 (7)*	
H2N2	0.0853 (15)	0.931 (2)	0.0958 (13)	0.032 (6)*	
H1N3	0.1265 (15)	0.539 (2)	0.0688 (13)	0.030 (6)*	
H2N3	0.0790 (17)	0.624 (2)	−0.0146 (15)	0.045 (6)*	

### Atomic displacement parameters (Å^2^)

**Table:**

	*U*^11^	*U*^22^	*U*^33^	*U*^12^	*U*^13^	*U*^23^
S1	0.0226 (2)	0.01564 (19)	0.01148 (17)	0.00260 (15)	0.00556 (15)	0.00000 (14)
S2	0.0387 (3)	0.0255 (2)	0.01363 (19)	0.0129 (2)	0.00060 (18)	−0.00185 (16)
O1	0.0260 (6)	0.0249 (6)	0.0173 (5)	0.0073 (5)	0.0123 (5)	0.0050 (5)
O2	0.0197 (6)	0.0224 (6)	0.0167 (5)	0.0031 (5)	0.0061 (4)	0.0002 (4)
O3	0.0423 (7)	0.0159 (6)	0.0162 (5)	0.0007 (5)	0.0034 (5)	−0.0021 (5)
N1	0.0212 (7)	0.0359 (8)	0.0135 (6)	−0.0095 (6)	0.0075 (6)	−0.0006 (6)
N2	0.0325 (8)	0.0175 (7)	0.0143 (7)	0.0026 (6)	0.0043 (6)	−0.0017 (6)
N3	0.0312 (8)	0.0168 (7)	0.0138 (6)	0.0035 (6)	0.0045 (6)	0.0005 (6)
C1	0.0231 (9)	0.0303 (9)	0.0184 (7)	−0.0017 (7)	0.0089 (7)	−0.0008 (7)
C2	0.0356 (10)	0.0353 (10)	0.0193 (8)	−0.0066 (9)	0.0102 (8)	−0.0068 (8)
C3	0.0282 (10)	0.0414 (11)	0.0207 (8)	−0.0135 (9)	0.0041 (7)	−0.0027 (8)
C4	0.0186 (8)	0.0371 (10)	0.0223 (8)	−0.0013 (8)	0.0060 (7)	0.0047 (7)
C5	0.0214 (8)	0.0246 (8)	0.0182 (7)	0.0015 (7)	0.0075 (6)	0.0013 (7)
C6	0.0198 (8)	0.0214 (8)	0.0130 (7)	−0.0009 (7)	0.0032 (6)	0.0032 (6)
C7	0.0191 (8)	0.0191 (8)	0.0139 (7)	−0.0006 (6)	0.0058 (6)	−0.0004 (6)
C8	0.0190 (8)	0.0198 (8)	0.0160 (7)	−0.0035 (7)	0.0053 (6)	0.0008 (6)
C9	0.0221 (8)	0.0183 (7)	0.0167 (7)	−0.0016 (7)	0.0091 (6)	0.0011 (6)
C10	0.0208 (8)	0.0149 (7)	0.0128 (6)	0.0026 (6)	0.0062 (6)	0.0008 (6)
C11	0.0186 (8)	0.0188 (8)	0.0162 (7)	−0.0019 (6)	0.0056 (6)	−0.0004 (6)
C12	0.0209 (8)	0.0234 (8)	0.0149 (7)	−0.0014 (7)	0.0078 (6)	0.0031 (6)
C13	0.0295 (9)	0.0212 (8)	0.0198 (8)	0.0024 (7)	0.0099 (7)	−0.0006 (7)
C14	0.0390 (11)	0.0283 (9)	0.0202 (8)	0.0112 (8)	0.0134 (8)	0.0051 (7)
C15	0.0343 (10)	0.0405 (11)	0.0160 (8)	0.0115 (9)	0.0029 (8)	−0.0023 (8)
C16	0.0279 (10)	0.0387 (11)	0.0238 (9)	0.0001 (9)	0.0028 (8)	−0.0087 (8)
C17	0.0252 (9)	0.0253 (9)	0.0220 (8)	−0.0001 (7)	0.0073 (7)	−0.0030 (7)
C18	0.0209 (8)	0.0195 (8)	0.0162 (7)	0.0054 (7)	0.0064 (6)	−0.0009 (6)
C19	0.0233 (9)	0.0189 (8)	0.0161 (7)	0.0021 (7)	0.0064 (6)	−0.0003 (6)
C20	0.0200 (8)	0.0204 (8)	0.0150 (7)	0.0006 (7)	0.0051 (6)	−0.0001 (6)

### Geometric parameters (Å, °)

**Table:**

S1—O2	1.4541 (12)	C5—H5	0.912 (18)
S1—O1	1.4612 (11)	C7—C8	1.397 (2)
S1—O3	1.4658 (12)	C7—C12	1.410 (2)
S1—C10	1.7474 (15)	C8—C9	1.387 (2)
S2—C20	1.7347 (16)	C8—H8	0.957 (19)
S2—C19	1.8208 (17)	C9—C10	1.387 (2)
N1—C7	1.3800 (19)	C9—H9	0.974 (18)
N1—C6	1.403 (2)	C10—C11	1.397 (2)
N1—H1N1	0.85 (2)	C11—C12	1.379 (2)
N2—C20	1.315 (2)	C11—H11	0.982 (17)
N2—H1N2	0.92 (3)	C12—H12	0.961 (18)
N2—H2N2	0.84 (2)	C13—C14	1.387 (2)
N3—C20	1.311 (2)	C13—C18	1.394 (2)
N3—H1N3	0.81 (2)	C13—H13	0.96 (2)
N3—H2N3	0.91 (2)	C14—C15	1.378 (3)
C1—C2	1.380 (2)	C14—H14	0.92 (2)
C1—C6	1.398 (2)	C15—C16	1.386 (3)
C1—H1	1.00 (2)	C15—H15	0.92 (2)
C2—C3	1.383 (3)	C16—C17	1.392 (2)
C2—H2	0.95 (2)	C16—H16	0.96 (2)
C3—C4	1.383 (3)	C17—C18	1.384 (2)
C3—H3	0.97 (2)	C17—H17	0.99 (2)
C4—C5	1.382 (2)	C18—C19	1.510 (2)
C4—H4	0.947 (19)	C19—H19A	0.955 (19)
C5—C6	1.389 (2)	C19—H19B	0.973 (18)
			
O2—S1—O1	112.06 (7)	C10—C9—C8	120.73 (14)
O2—S1—O3	111.18 (7)	C10—C9—H9	120.8 (11)
O1—S1—O3	111.84 (7)	C8—C9—H9	118.5 (11)
O2—S1—C10	107.66 (7)	C9—C10—C11	119.74 (14)
O1—S1—C10	106.02 (7)	C9—C10—S1	119.82 (11)
O3—S1—C10	107.76 (7)	C11—C10—S1	120.28 (12)
C20—S2—C19	106.38 (8)	C12—C11—C10	119.63 (15)
C7—N1—C6	128.35 (14)	C12—C11—H11	120.9 (10)
C7—N1—H1N1	113.6 (13)	C10—C11—H11	119.5 (10)
C6—N1—H1N1	116.2 (13)	C11—C12—C7	121.22 (14)
C20—N2—H1N2	117.4 (16)	C11—C12—H12	119.7 (11)
C20—N2—H2N2	122.1 (14)	C7—C12—H12	119.1 (11)
H1N2—N2—H2N2	120 (2)	C14—C13—C18	120.14 (17)
C20—N3—H1N3	118.7 (14)	C14—C13—H13	118.8 (12)
C20—N3—H2N3	121.6 (14)	C18—C13—H13	121.1 (12)
H1N3—N3—H2N3	120 (2)	C15—C14—C13	120.46 (18)
C2—C1—C6	120.70 (16)	C15—C14—H14	118.7 (12)
C2—C1—H1	121.4 (11)	C13—C14—H14	120.9 (13)
C6—C1—H1	117.9 (11)	C14—C15—C16	119.74 (17)
C1—C2—C3	120.23 (17)	C14—C15—H15	121.8 (14)
C1—C2—H2	118.2 (13)	C16—C15—H15	118.4 (14)
C3—C2—H2	121.6 (13)	C15—C16—C17	120.03 (18)
C4—C3—C2	119.27 (17)	C15—C16—H16	119.3 (12)
C4—C3—H3	121.7 (12)	C17—C16—H16	120.7 (12)
C2—C3—H3	118.9 (11)	C18—C17—C16	120.40 (17)
C5—C4—C3	120.97 (17)	C18—C17—H17	120.7 (11)
C5—C4—H4	119.0 (12)	C16—C17—H17	118.9 (11)
C3—C4—H4	120.0 (12)	C17—C18—C13	119.20 (15)
C4—C5—C6	120.13 (16)	C17—C18—C19	120.36 (15)
C4—C5—H5	119.3 (12)	C13—C18—C19	120.44 (15)
C6—C5—H5	120.6 (12)	C18—C19—S2	105.09 (11)
C5—C6—C1	118.68 (15)	C18—C19—H19A	112.0 (11)
C5—C6—N1	123.27 (15)	S2—C19—H19A	106.8 (11)
C1—C6—N1	118.01 (15)	C18—C19—H19B	112.3 (11)
N1—C7—C8	124.05 (15)	S2—C19—H19B	108.1 (11)
N1—C7—C12	117.57 (14)	H19A—C19—H19B	112.0 (15)
C8—C7—C12	118.37 (14)	N3—C20—N2	121.75 (15)
C9—C8—C7	120.28 (15)	N3—C20—S2	114.84 (12)
C9—C8—H8	117.7 (11)	N2—C20—S2	123.36 (12)
C7—C8—H8	121.9 (11)		
			
C6—C1—C2—C3	0.0 (3)	O1—S1—C10—C11	−174.82 (12)
C1—C2—C3—C4	−0.9 (3)	O3—S1—C10—C11	−54.93 (15)
C2—C3—C4—C5	0.3 (3)	C9—C10—C11—C12	0.9 (2)
C3—C4—C5—C6	1.2 (3)	S1—C10—C11—C12	−174.38 (12)
C4—C5—C6—C1	−2.1 (3)	C10—C11—C12—C7	0.1 (2)
C4—C5—C6—N1	−179.60 (16)	N1—C7—C12—C11	177.74 (15)
C2—C1—C6—C5	1.6 (3)	C8—C7—C12—C11	−1.5 (2)
C2—C1—C6—N1	179.16 (16)	C18—C13—C14—C15	1.1 (3)
C7—N1—C6—C5	−46.7 (3)	C13—C14—C15—C16	0.0 (3)
C7—N1—C6—C1	135.84 (18)	C14—C15—C16—C17	−1.2 (3)
C6—N1—C7—C8	4.1 (3)	C15—C16—C17—C18	1.3 (3)
C6—N1—C7—C12	−175.08 (16)	C16—C17—C18—C13	−0.2 (3)
N1—C7—C8—C9	−177.33 (16)	C16—C17—C18—C19	−179.89 (16)
C12—C7—C8—C9	1.8 (2)	C14—C13—C18—C17	−1.0 (2)
C7—C8—C9—C10	−0.8 (2)	C14—C13—C18—C19	178.70 (15)
C8—C9—C10—C11	−0.6 (2)	C17—C18—C19—S2	118.32 (15)
C8—C9—C10—S1	174.76 (12)	C13—C18—C19—S2	−61.34 (17)
O2—S1—C10—C9	−110.24 (13)	C20—S2—C19—C18	−177.78 (11)
O1—S1—C10—C9	9.86 (15)	C19—S2—C20—N3	−167.79 (13)
O3—S1—C10—C9	129.75 (13)	C19—S2—C20—N2	14.60 (17)
O2—S1—C10—C11	65.08 (14)		

### Hydrogen-bond geometry (Å, °)

**Table:**

*D*—H···*A*	*D*—H	H···*A*	*D*···*A*	*D*—H···*A*
N1—H1N1···O1^i^	0.85 (2)	1.99 (2)	2.836 (2)	173.0 (18)
N2—H1N2···O3^ii^	0.92 (3)	2.04 (3)	2.9561 (19)	172 (3)
N3—H1N3···O1	0.809 (19)	2.015 (19)	2.8204 (19)	174 (2)
N2—H2N2···O3^iii^	0.839 (19)	2.015 (19)	2.8069 (19)	157.2 (19)
N3—H2N3···O2^ii^	0.91 (2)	1.96 (2)	2.8633 (17)	173 (2)
C9—H9···O1	0.97 (2)	2.463 (18)	2.8563 (18)	103.8 (13)
C19—H19B···O2^iv^	0.97 (2)	2.57 (2)	3.332 (2)	134.8 (14)
C4—H4···Cg2^v^	0.947 (19)	3.22 (2)	3.943 (2)	134.7 (14)
C17—H17···Cg1^iii^	0.99 (2)	2.92 (2)	3.471 (2)	116.0 (15)

Symmetry codes: (i) *x*, −*y*+1/2, *z*+1/2; (ii) −*x*, −*y*+1, −*z*;  (iii) *x*, *y*+1, *z*; (iv) −*x*, *y*+1/2, −*z*+1/2; (v) −*x*+1, −*y*+1, −*z*+1.
